# The impact of manual vacuum-assisted closure technique in wound healing: a case report

**DOI:** 10.1186/s13256-023-04306-0

**Published:** 2024-01-23

**Authors:** Hema Malini, Ismiati Ismiati, Wrisma Arif Harahap, Elvi Oktarina

**Affiliations:** Faculty of Nursing, Universitas Andalas, Padang City, West Sumatera, 25163 Indonesia

**Keywords:** Foot ulcer, Diabetic, Wound healing, Debridement, Case report

## Abstract

**Background:**

Diabetic ulcers are complex wounds that require specialized care. Proper wound care is crucial to prevent amputation, and one effective treatment option is negative pressure wound therapy. However, the cost of negative pressure wound therapy can often be a barrier, making it difficult for caregivers and families to access.

**Aim:**

This study aims to develop an alternative system, called the manual vacuum-assisted closure technique, using a 50 cc syringe pump with a pressure value of 93.33 mmHg, to examine the impact of the manual vacuum-assisted closure technique on the continuum of wound status in diabetic ulcers.

**Case presentation:**

A 56-year-old Minangnese man, with a 15-year history of diabetes mellitus and a family history of the disease, presented with a grade IV diabetic ulcer on the dorsal pedis dextra following a postoperative debridement. The wound measured 48 cm^2^ and had an ankle–brachial index value of 1.0 mmHg. The ulcer originated from being pierced by a nail. Previous treatment involved surgical debridement in early January, followed by twice-daily wound care using gauze and 0.9% NaCl, which showed no improvement. Consequently, the wound worsened and became more painful. The patient also had a history of smoking, which he only quit earlier this year. The wound was assessed using the Bates–Jensen Wound Assessment Tool over a period of 21 days.

**Conclusion:**

After daily manual vacuum-assisted closure technique wound treatment for 21 days in diabetic ulcers, there was a noticeable decrease in the Bates–Jensen Wound Assessment Tool scores. Specifically, on day 5, the score was 38; on day 14, the score was 30; and on day 21, the score was 24. The use of the manual vacuum-assisted closure technique in wound treatment demonstrated significant improvements in diabetic ulcers.

## Introduction

A diabetic ulcer is one of the macrovascular complications of type II diabetes mellitus, which is caused by peripheral neuropathy [8]. The prevalence of patients with diabetic ulcers in Indonesia is around 15%, with an amputation rate of 3% and a mortality rate of 14.8% 1 year after amputation [6]. Data from Balitbangkes Kemenkes [2] show that the number of people who suffer from diabetic ulcers in Indonesia has increased by 11%.

Diabetic ulcers are open wounds on the surface of the skin caused by macroangiopathy, resulting in vascular inducibility, neuropathy, vasculopathy, immunopathy, and foot biomechanics [7]. Consequently, they require prolonged healing time, leading to considerable treatment costs that can negatively impact the economy, the quality of life, and even increase morbidity and mortality. With rapid technological advances, wound care methods using the moisture balance principle, such as negative pressure wound therapy (NPWT), have become more effective in wound healing [5].

NPWT is increasingly used in managing diabetic foot ulcers with compensated peripheral circulation, reducing exudate, and promoting wound-induced macro- and microdeformations, along with increased granulation and tissue proliferation [5]. However, the cost of the NPWT machine and disposable foam dressings poses challenges in using this method. To address this issue, we developed an economical tool using the NPWT principle, known as the manual vacuum-assisted closure technique (MVACT).

**Fig. 1:**
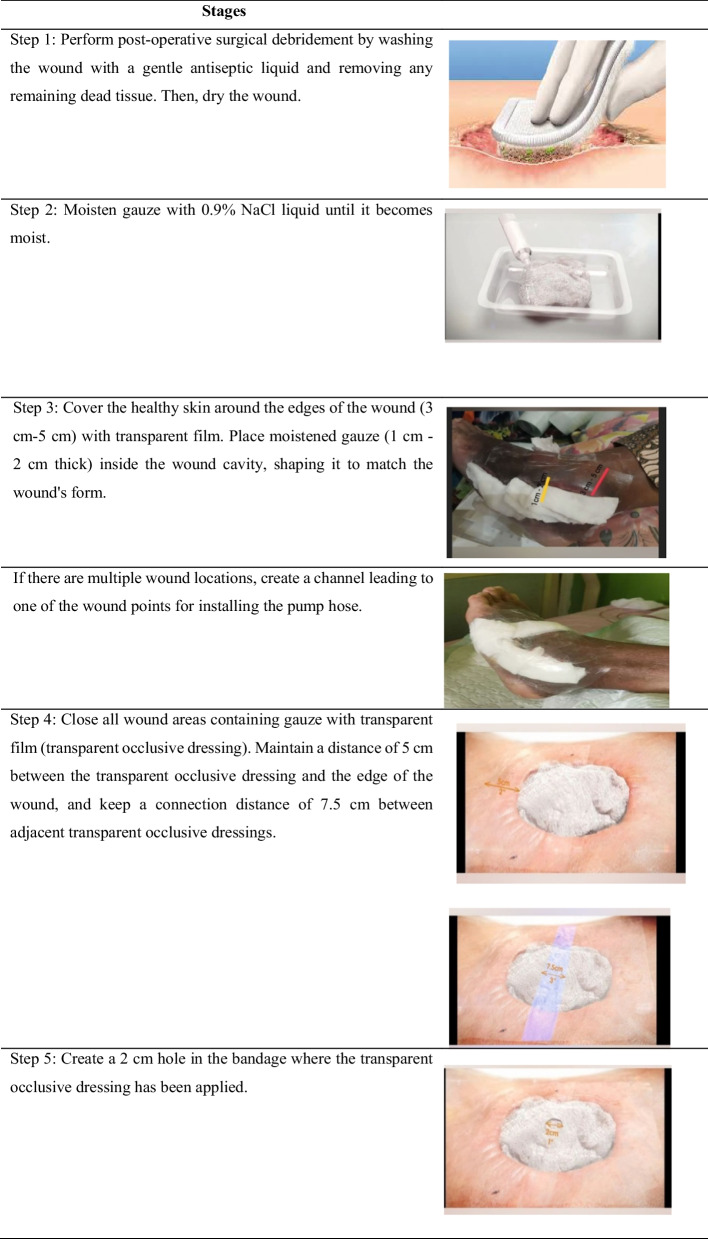

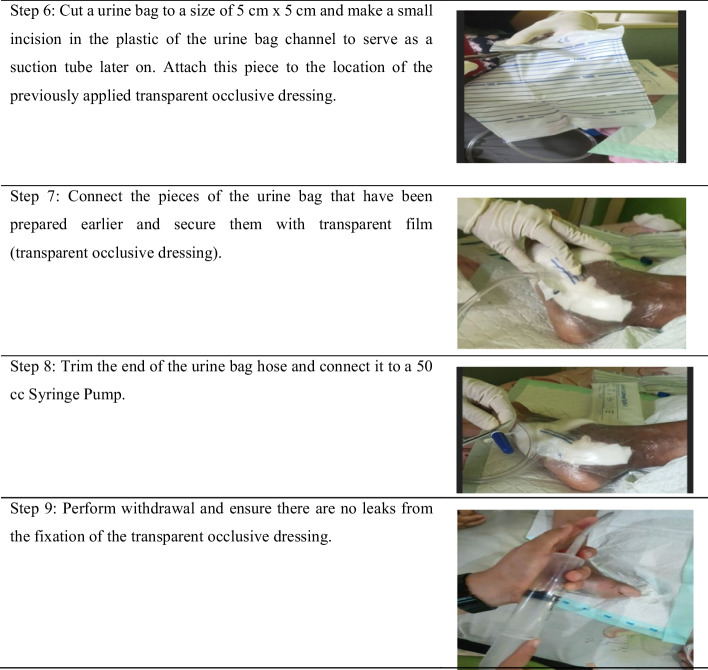
Application of the manual vacuum.assisted closure technique

The MVACT modifies the vacuum device’s principle to provide a simple and easy-to-use noninvasive treatment modality utilizing controlled negative pressure with a pressure value of 93.33 mmHg. It employs a vacuum-assisted closure device or manually closed suction system to accelerate wound healing by removing excess fluid from wounds, reducing edema, and promoting granulation tissue formation. To measure the wound status continuum, this study uses the Bates–Jensen Wound Assessment Tool (BJWAT) [10]. This case study reports the impact of the manual vacuum-assisted closure technique on wound healing (Fig. 1).

## Case presentation

This wound-care case study, using MVACT, demonstrates tissue changes in several components of wound assessment. These changes include reduced wound size, depth, granulation percentage, epithelialization, necrotic tissue, and multiple sloughs. The initial assessment was conducted on a self-employed 56-year-old Minangnese man with a high school education and a 15-year history of diabetes mellitus. There is also a family history of diabetes mellitus. The patient presented with a postdebridement wound on the first day, which was diagnosed as a diabetic ulcer on the dorsal pedis dextra resulting from a nail puncture. Prior to the study, surgical debridement was performed 5 months ago, and wound treatment involved using gauze and 0.9% NaCl twice a day, but no improvement was observed. Over time, the wound extended and became more painful. Additionally, the patient had a history of smoking, which he recently quit. The ankle–brachial index (ABI) value was measured at 1.0 mmHg. The day 1 assessment, using the BJWAT, revealed a score of 43 (Fig. 2.1–2.4).

**Fig. 2:**
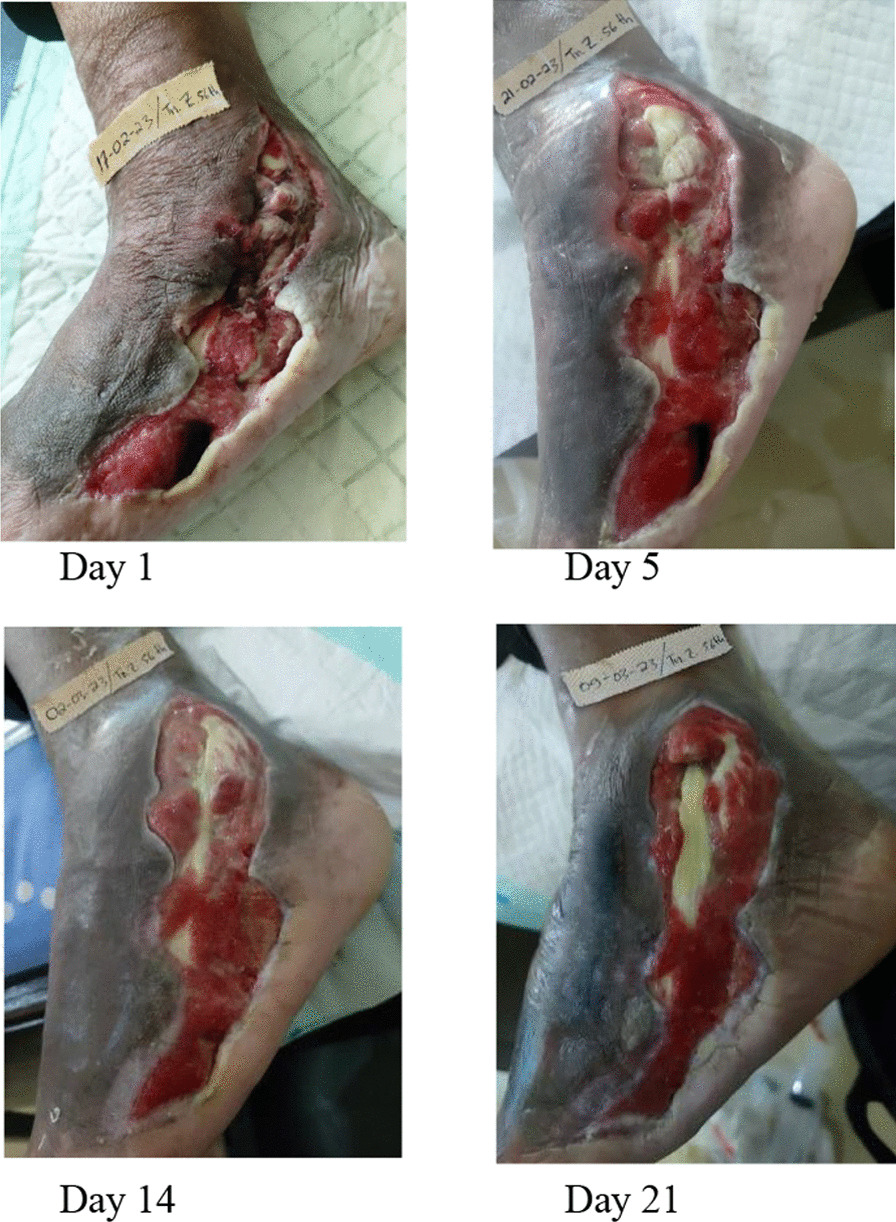
The development of BWAT score from day 1-21

After 21 days of wound treatment with MVACT, there was a noticeable decrease in the BJWAT scores. Specifically, on day 5, the score was 38 (Fig. 2.2); on day 14, the score was 30 (Fig. 2.3); and on day 21, the score was 24 (Fig. 2.4).

## Discussion

This case report presents the effect of wound treatment with MVACT on diabetic ulcer wound healing. The discussion focuses on macrodeformation, microdeformation, and exudate control, adjusted to the measurement of the BJWAT.

Macrodeformation refers to tissue deformation resulting from centripetal withdrawal at the edge of the wound. The effects of macrodeformations vary depending on the depth of the wound tissue. The superficial part of the wound experiences compressive force, leading to hypoperfusion, while the deep part is subjected to traction forces, resulting in dilation of blood vessels and hyperperfusion. Superficial hypoperfusion and hypoxia create a gradient of vascular endothelial growth factors that promote angiogenesis, while deep hyperperfusion improves nutrient and oxygen delivery (Fig. 2.2–2.4). The effect of MVACT on macrodeformation was observed on day 5 after insertion, where the wound edge fused with the wound base. On days 14–21, the wound size was reduced by greater than 50%, the cave size became smaller, and the wound color turned red like flesh, indicating improved wound healing.

Microdeformation is also crucial in the efficacy of NPWT in cell proliferation and increased granulation tissue production. NPWT induces tissue microdeformation at the wound base, causing cell stretching and subsequent cell proliferation, leading to angiogenesis and granulation tissue formation. Microvascular mechanisms observed in this case include wound size reduction (from grade IV to grade II) due to accelerated granulation and epithelialization processes (> 50% and > 25%, respectively) on day 14 [4].

NPWT exerts mechanical force and can remove excess interstitial fluid, increase tissue pressure, reduce edema, and facilitate healing in acute and closed wounds. NPWT also removes potentially toxic components from chronic wound exudate, benefiting chronic wounds. In the study by Anjum *et al*. [1], NPWT resulted in reduced wound volume and depth by greater than 25% and increased granulation tissue by 50%.

MVACT, based on NPWT principles, can also reduce exudate and control wound infection, thereby promoting faster wound healing. MVACT creates a moist atmosphere, which utilizes atmospheric pressure to expedite the healing process. The impact of MVACT on wound healing is similar to that of NPWT. One case study [3] showed that wound treatment using NPWT in patients with diabetic ulcers achieved granulation in 75–100% of cases. This finding is consistent with the study by Seidel *et al*. [9], which demonstrated accelerated epithelialization and a shorter time to wound closure in patients treated with NPWT.

Participants in this study reported minimal pain during the MVACT procedure and observed rapid improvement in wound condition, reduced odor, and a sense of cleanliness due to effective exudate management through the applied wound dressing. Changing drese effortless, expediting wound bed preparation for granulation.

## Conclusion

Over 3 weeks, this case study showed a decrease in BJWAT scores after wound treatment using MVACT. MVACT treatment appears to be a promising alternative for diabetic ulcer wound care, leading to improved patient outcomes and reduced adverse complications such as odor, pain, and further infection.

## Data Availability

Not applicable.

## References

[1] Anjum W, Ali SZ, Mumtaz M, Imran M, Siddique H, Zia H (2022). Comparison of vacuum assisted closure (VAC) therapy versus conventional dressing in the management of diabetic foot ulcer. Pak J Med Health Sci.

[2] Balitbangkes Kemenkes. Infodatin tetap produktif, cegah, dan atasi Diabetes Melitus 2020. In: Pusat Data dan Informasi Kementerian Kesehatan RI 2020; pp. 1–10.

[3] Borys S, Hohendorff J, Frankfurter C, Kiec-Wilk B, Malecki MT (2019). Negative pressure wound therapy use in diabetic foot syndrome-from mechanisms of action to clinical practice. Eur J Clin Invest.

[4] Horch RE, Ludolph I, Müller-Seubert W, Zetzmann K, Hauck T, Arkudas A, Geierlehner A (2020). Topical negative-pressure wound therapy: emerging devices and techniques. Expert Rev Med Devices.

[5] Kim PJ, Attinger CE, Olawoye O, Crist BD, Gabriel A, Galiano RD, Gupta S, Lantis JC, Lavery L, Lipsky BA, Teot L (2015). Negative pressure wound therapy with instillation: review of evidence and recommendations. Wounds.

[6] Ministry of Health RI. Hasil Utama LaporanRiskesdas 2018. 2018.

[7] Pitocco D, Spanu T, Di Leo M, Vitiello R, Rizzi A, Tartaglione L, Fiori B, Caputo S, Tinelli G, Zaccardi F, Flex A, Galli M, Pontecorvi A, Sanguinetti M (2019). Diabetic foot infections: a comprehensive overview. Eur Rev Med Pharmacol Sci.

[8] Rangel ÉB, Rodrigues CO, De Sá JR (2019). Micro- and macrovascular complications in diabetes mellitus: preclinical and clinical studies. J Diabetes Res.

[9] Seidel D, Storck M, Lawall H, Wozniak G, Mauckner P, Hochlenert D, Wetzel-Roth W, Sondern K, Hahn M, Rothenaicher G, Krönert T, Zink K, Neugebauer E (2020). Negative pressure wound therapy compared with standard moist wound care on diabetic foot ulcers in real-life clinical practice: results of the German DiaFu-RCT. BMJ Open.

[10] sralab.org. Bates-Jensen Wound Assessment Tool. 2019. https://www.sralab.org/rehabilitation-measures/bates-jensen-wound-assessment-tool

